# New Advances in Reproductive Biomedicine

**DOI:** 10.1155/2014/529170

**Published:** 2014-06-19

**Authors:** Irma Virant-Klun, Jeroen Krijgsveld, John Huntriss, Raymond J. Rodgers

**Affiliations:** ^1^Reproductive Unit, Department of Obstetrics and Gynaecology, University Medical Centre Ljubljana, Slajmerjeva 3, 1000 Ljubljana, Slovenia; ^2^Genome Biology Unit and Proteomics Core Facility, European Molecular Biology Laboratory, Meyerhofstraße 1, 69117 Heidelberg, Germany; ^3^Division of Reproduction and Early Development, Leeds Institute of Genetics, Health and Therapeutics, The LIGHT Laboratories, University of Leeds, Clarendon Way, Leeds, UK; ^4^Research Centre for Reproductive Health, Discipline of Obstetrics and Gynaecology, School of Paediatrics and Reproductive Health, Robinson Institute, The University of Adelaide, Adelaide, SA, Australia

Since birth of the first “test tube” baby—Louise Brown—in 1978, pioneered by physiologist Robert G. Edwards and surgeon Patrick C. Steptoe, there was a great advance in reproductive biomedicine made by amazing people, who insisted on this path in spite of pressure from society. In vitro fertilization procedure indeed became an established way to treat couples with severe infertility problems, and until today more than four million babies have been born worldwide in this way. The field of assisted conception is a fast-developing domain at both the basic and clinical level. This is reflected in the 31 papers in this special issue, spanning a wide range of topics including female and male infertility, gametes, embryos, pregnancy, and babies born after assisted conception.

There are some new aspects to study and think about female (in)fertility. The methodology of proteomics was exposed to be important in potential linking oxidative stress and female infertility (S. Gupta et al.). One of the main tasks in the in vitro fertilization program remains to better elucidate oocyte quality. This can be facilitated by novel proteomic and secretome tools as have been used in animal models, although some obstacles still need to be resolved before these can be applied to human cells (I. Virant-Klun and J. Krijgsveld). The quality of oocytes also depends on mutual interactions with surrounding granulosa cells; therefore, global profiling of gene expression of follicular cumulus cells using microarrays and next generation sequencing (NGS) may lead to establishment of new reliable biomarkers to select oocytes with the highest developmental potential (E. Chronowska). Oocyte maturity is a prerequisite for successful fertilization and pregnancy. Time-lapse dynamics of oocyte chromatin organisation during meiotic resumption was observed in a mouse model, providing a pathway of transition from the GV to the MII stage oocyte (M. Belli et al.). In vitro maturation (IVM) of human oocytes is still a suboptimal procedure resulting in impaired oocyte developmental potential. However, results on bovine oocytes showed that addition of hyaluronan (HA) to the IVM medium improved the quality of in vitro derived embryos-blastocysts, which expressed less DNA fragmentation (J. Opiela et al.). Oocyte quality can be influenced by female infertility factors. In nonoverweight women with polycystic ovary syndrome (PCOS), the association between follicular fluid leptin and serum insulin was confirmed (G. Garruti et al.). It was proposed that lower levels of leptin in follicular fluid may explain the lower oocyte quality and fertilization in these women and this may be related to the level of insulin and/or insulin resistance.

Most instances of severe infertility in men cannot be explained. A special concern is represented by male and couple fertility impairment due to human papilloma virus (HPV) DNA sperm infection (S. Gizzo et al.). The question of potential negative impact of HPV infection on semen quality is still open, despite high prevalence of infection (37.1%) in men from infertile couples (B. Golob et al.). In nonobstructive azoospermia, the increase in volume density of both the CD 68-positive macrophages and vacuolated Leydig cells and a positive correlation between the volume densities of these cell types were observed; it was proposed that overstimulation of Leydig cells, together with a negative paracrine action of macrophages, could result in the damage of steroidogenesis and lower levels of testosterone in situ (T. Goluza et al.). The fertilization potential of spermatozoa depends on appropriate and time-dependent process of hyperactivation, chemotaxis, capacitation, and the acrosome reaction, where calcium (Ca^2+^) is extensively involved in almost every step. Modern proteomic analyses have identified several sperm proteins, and therefore these findings might provide further insight into understanding the Ca^2+^ influx, protein functions, and regulation of fertility (M. S. Rahman et al.).

At present there is still no generally accepted clinical treatment to improve the semen quality in infertile men, although some clinical trials showed that this may be improved by oral administration of antioxidants (S. Imamovic Kumalic and B. Pinter). Furthermore, a special concern are also the paternal, sperm-borne mitochondria (mt) that are selectively degraded inside the fertilized oocytes. Assisted reproductive therapies (ART) such as intracytoplasmic sperm injection (ICSI) for male infertility treatment include the injection of the entire sperm into oocytes including mitochondria and mtDNA; therefore, sperm mitochondrial degradation mechanisms may play a crucial role with implications to health, fitness and fertility, and development and health of ART babies (W.-H. Song et al.).

Array comparative genomic hybridization (aCGH) for comprehensive screening of all chromosomes is becoming a new tool for selection of euploid embryos to be transferred in the in vitro fertilization program (L. Rodrigo et al.) and can improve the clinical outcome—pregnancy rate—in women with repeated implantation failure (E. Greco et al.). Yet, some caution is needed, because embryo biopsy is an invasive procedure, and less invasive procedures to distinct normal and abnormal embryos need to be developed (T. Vladimirova Milachich). Not only embryo but also the endometrium plays an important role in successful implantation. One of the first studies confirmed that the infusion of granulocyte colony-stimulating factor (G-CSF) leads to the improvement in endometrium thickness and the chance to achieve pregnancy in women with treatment-resistant thin endometrium (M. Kunicki et al.). In a clinical study, tissue Doppler imaging (TDI) based elastography was used for assessment of cervical stiffness during all three trimesters of pregnancy and a correlation with gestational age, cervical length, and parity was found (A. Fruscalzo et al.). Fetal middle cerebral artery (MCA) circulation was followed after defined prenatal acoustical stimulation (PAS), correlating the role of cilia in hearing and memory in fetus to signal transduction (i.e., optical-acoustical properties). The authors suggest that fetuses are getting used to sound, developing a kind of memory patterns, considering acoustical and electromagnetic waves (S. Jankovic-Raznatovic et al.). Further, BACs-on-beads technology represents a new and reliable test for rapid detection of fetal aneuploidies and microdeletions in prenatal diagnostics (S. García-Herrero et al.). Quantitative proteomics analysis revealed the altered protein expression in the placental villous tissue of early pregnancy loss using isobaric tandem mass tags and may represent a new diagnostic tool in the future (X. Ni et al.).

Besides in vitro fertilization itself, cryopreservation of embryos, gametes, and reproductive tissues represents an extremely important part of assisted conception to preserve the potential of life and fertility, as reviewed by J. Konc et al. Advanced experimental data were provided on new approaches to human ovarian tissue cryopreservation in young cancer patients, showing vitrification with good preservation of stromal cells without apoptosis in human ovarian tissue (R. Fabbri et al.) and an effective in vitro perfusion of ovine intact ovaries with vascular pedicle by freezing medium, important for development of whole ovary cryopreservation in humans (V. Isachenko et al.). An option in cancer patients is also to cryopreserve the oocytes, if ovarian stimulation is safe and possible, but this is not an easy task and was thoroughly reviewed in bovine oocytes (I.-S. Hwang and S. Hochi). The data also show that superoxide dismutase (SOD) represents a good predicting factor for boar semen characteristics after short-term cryostorage (M. Zakosek Pipan et al.) and may be applied in human medicine.

Each birth of a baby after in vitro fertilization still represents a great effort and a “miracle.” During the last years, several studies have shown that babies after assisted conception are healthy and do not differ from other babies. Nevertheless, permanent concern is needed for both mother and baby to be safe and healthy. In a clinical study, singleton pregnancy and neonate outcomes after in vitro fertilization with fresh or frozen-thawed embryo transfer were followed in a large group of women (S. Korosec et al.).

The perspectives and challenges of pluripotent and induced pluripotent stem cells as new agents for treatment of infertility are discussed by V. Volarevic et al. Moreover, stem cells from follicular aspirates in infertile women were successfully cultured in vitro. They expressed several genes related to mesenchymal stem cells and were successfully differentiated into cells of all three germ layers (E. Dzafic et al.). These cells may be interesting for both the reproductive (e.g., regeneration of ovaries) and regenerative medicine. Also spermatogonial stem cells were further researched by generating gene expression profiles for human spermatogonia both after short- and long-term culture (S. Conrad et al.).

In this special issue, it is also reported that full reimbursement of in vitro fertilization with repeated attempts can provide a notable success considering cumulative delivery rate and should thus be proposed to public health systems (U. Vrtacnik et al.). All great efforts summarized in this special issue not only bring several scientific and clinical answers but also open some new questions, which are an important task and an exciting challenge for the future.

*Irma Virant-Klun* *Jeroen Krijgsveld* *John Huntriss* *Raymond J. Rodgers*

